# Judicial Actors’ Understanding of the Mental Health Impacts of Intimate Partner Violence: A Scoping Review

**DOI:** 10.1177/15248380241244494

**Published:** 2024-04-17

**Authors:** Susan Lynn Heward-Belle, Parveen Azam Ali, Julieta Marotta, Debbie Hager, Michaela Rogers, Lynette Stevenson

**Affiliations:** 1 University of Sydney, NSW, Australia; 2 University of Sheffield, UK; 3 Maastricht University, Limburg, Netherlands; 4 University of Auckland, New Zealand

**Keywords:** criminal justice, domestic abuse, intimate partner violence, legal framework, mental distress, victims, survivors

## Abstract

Intimate partner violence (IPV) is a global public health issue that has grave physical and mental health consequences for millions of women. The judicial system plays a critical role in responding to IPV principally through the criminal justice system, family law, and/or child welfare jurisdictions. However, victims/survivors who interact with the legal system report negative experiences. An under-researched area of scholarship is the degree to which judicial actors understand the mental health impacts of IPV on victims/survivors and how they apply that knowledge in practice. This scoping review aimed to identify and synthesize existing scholarship on judicial actors’ understanding of the mental health impacts of IPV on women survivors. We searched 10 databases (Medline, Scopus, PubMed, PsycINFO, EMBASE, Westlaw, HeinOnline, the Cochrane Library, and the Joanna Briggs Library databases) for studies published between 2000 and 2023. A total of 27 studies were included in the review. We identified five main themes, including: awareness of survivors’ experiences, gap in judicial actors’ knowledge, understanding of perpetrator tactics and risk factors, disclosing mental health problems, training, and guidance. The review highlights significant gaps in judicial actors’ understanding of this issue and recommends strategies to increase the awareness and understanding of IPV among judicial actors. The findings can be used to justify future research to better understand the training and development needs of judicial actors to improve their level of awareness of the dynamics and impact of IPV and to make policy and practice recommendations to build the capacity of the judicial workforce.

## Introduction

The legal system of any country plays a critical role in the lives of victim/survivors who may be seeking protection from abuse, assistance with separation, or divorce and/or custody of children. Intimate partner violence (IPV) is the most common manifestation of violence against women. It is a significant global public health issue that results in serious morbidity and mortality (World Health Organization, 2019). IPV refers to behavior by a current or previous intimate partner that causes physical, sexual, or psychological harm, including physical aggression, sexual coercion, psychological abuse, and controlling behavior (World Health Organization, 2019). IPV can occur within all types of relationships, does not require sexual intimacy, and can happen to people of any gender or sexual orientation; however, most victims remain cisgender women (Brown & James, 2014). The abuse they experience is often repeated, systematic, and has serious consequences.

Evidence from organizations that work with survivors suggests that the common mental health impacts that they experience because of IPV are either not well-understood by judicial actors or are often decontextualized and used by the courts to refuse protection orders for women, award child custody to abusive men, and/or remove children to live with relatives or others (Mackenzie & Herbert, 2017). Judicial actors here are defined as people who participate in the administration of justice, including judges, prosecutors, lawyers, and other court staff. IPV survivors may be involved in various legal jurisdictions such as criminal justice, family law, child welfare, and civil law.

Worldwide, it is estimated that one in three women experience either physical and/or sexual IPV or non-partner sexual violence in their lifetime (Sardinha et al., 2022). Ascertaining the actual prevalence of IPV is difficult due to underreporting, varying definitions of IPV, cultural taboos, and a range of study methods used to capture data (Ali et al., 2015; Sardinha et al., 2022). In addition, recall bias and the self-reporting nature of IPV can result in under-representation of the extent of IPV (Saberi et al., 2017). This causes significant variation in estimated lifetime prevalence of IPV in different regions of the world with higher prevalence rates in low-income and middle-income countries and regions than high-income countries (Sardinha et al., 2022).

Although there are physical, sexual, financial, and social consequences of IPV, it is the psychological and mental health effects that are commonly reported by women to be most impactful on their lives. These include emotional distress, suicidal ideation, suicide attempts, sustained fear, low self-esteem, stress-related headaches, obsessive-compulsive disorder, post-traumatic stress disorder (PTSD), disassociation, sleep disorders, shame, guilt, and self-mutilation, as well as related behaviors such as substance abuse and eating disorders (Chandan et al., 2020; Ogbe et al., 2020). Results of a cohort study conducted in the United Kingdom (Chandan et al., 2020) suggests a strong association between IPV exposure and mental illness with an adjusted incidence rate ratio of 2.77 (95% CI [2.58, 2.9]). Ayre et al. (2016) found that women survivors of IPV had a higher risk of experiencing mental health issues with anxiety disorders (35%) depressive disorders (32%), and self-inflicted injuries (19%). The physical, sexual, and psychological effects of IPV tax women’s resources, make it necessary, and difficult- for many women- to seek protection through the legal system for themselves and their children.

Judicial actors who do not understand the mental health impact of IPV on women may create an environment where “secondary victimization” of women can occur (Laing, 2017). Secondary victimization occurs when court processes mimic perpetrator patterns, reinforcing unequal power dynamics resulting in the potential to re-traumatize survivors. Herman (2015) described the impact of the adversarial court system stating:

The legal system is designed to protect men from the superior power of the state but not to protect women or children from the superior power of men. . . . If one set out by design to devise a system for provoking intrusive post-traumatic symptoms, one could not do better than a court of law. (p. 91).

A quarter of a century later, the relationship between IPV and mental distress remains unrecognized by judicial actors, often compounding the trauma of IPV. It is thus critical that judicial actors understand the impact that IPV can have on survivors’ mental health (Breiding et al., 2015; Lipsky & Caetano, 2009) and the role that legal processes may play in retraumatizing women.

It is also important for judicial actors to be aware of, and address, interlocking forms of oppression that can compromise survivors’ ability to seek safety and justice through the legal system. Women survivors of IPV who experience mental health problems and have intersecting aspects of identity that are marginalized face additional disadvantages in the legal system. For example, survivors from black and minority ethnic communities, those from culturally and linguistically diverse backgrounds, those living with disabilities, or in rural and/or remote locations, who have been incarcerated, who are from lesbian, gay, bisexual, or trans communities, or who are economically marginalized are more likely to receive a poor legal response (Day & Gill, 2020; Frohmader et al., 2015; Stiles-Shields & Carroll, 2015). This scoping review seeks to explore judicial actors’ understanding of the mental health impacts of IPV on women survivors and in relation to women from diverse communities and background. We seek to answer the following research questions to guide future research and policy recommendations:

1. What is the scope and key findings of existing research exploring judicial actors’ understanding of the mental health impacts of IPV and/or survivors’ experience of judicial actors’ understanding of the mental health impacts of IPV?
2. What strategies and recommendations have been made to increase judicial actors’ awareness and understanding of the mental health impacts of IPV on survivors?

## Method

We undertook a scoping review—a systematic and iterative approach to identify and synthesize an existing or emerging body of literature on a given topic. Although there are several reasons for conducting a scoping review, the main reasons are to map the extent, range, and the nature of the literature, as well as to determine gaps in the literature on a given topic (Mak & Thomas, 2022). The guidance on scoping reviews by Peters et al. (2020) was used to ensure integrity and robustness of all aspects of the review including question formulation; inclusion and exclusion criteria articulation; development of a replicable search strategy with a decision flowchart and data extraction.

### Eligibility Criteria

Any empirical study that explored judicial actors’ understanding of the mental health impacts of IPV on survivors, or survivor’s experiences and perspectives about judicial actors’ understanding of the issue, and/or recommendations to increase judicial actors’ understanding of the mental health impact of IPV were considered. For inclusion, studies had to be (a) based on empirical data (quantitative, qualitative, or mixed methods); (b) written in English; (c) published in a peer-reviewed journal; and (d) published during the period August 2000–July 2023. Scholarly or theoretical papers, editorials, commentaries, and articles published in any language other than English were excluded from the review.

### Data Sources

A comprehensive literature search using PubMed, Scopus, MEDLINE, PsycINFO, Excerpta Medica Database (EMBASE), Westlaw, HeinOnline, the Cochrane Library, and the Joanna Briggs Library databases was performed. These databases were chosen to ensure that all appropriate evidence from the fields of health, law, and the social sciences were included. Keywords used in the search included IPV, judicial actors, judges, lawyers, mental health, mental disorders, mental illness, psychiatry*, psychology*, and trauma. Using Boolean operators in combination with broader terms enabled a comprehensive exploration of the search engines. A search was also conducted using Google and Google Scholar to identify studies not published in indexed journals. In addition, the reference list of each article was scrutinized to identify studies that may not have been listed in the searched databases. Box 1 lists the full search terms and Boolean operator combinations used for all key concepts. Use of search terms and keywords was kept consistent for all databases.

**Box 1.** Database search terms and keywords

Searches were conducted within *PubMed, Scopus, MEDLINE, PsycINFO, Excerpta Medica Database (EMBASE), Westlaw, HeinOnline*, the *Cochrane Library* and the *Joanna Briggs Library* databases using combinations of the following search terms and Boolean operator combinations: (“battered women” OR “domestic violence” OR “intimate partner violence” OR “domestic abuse” OR “family abuse” OR “family violence” OR “spousal assault” OR “spous* abuse”) AND (“mental health” OR “mental illness” OR “mental disorders” OR psychiatr* OR “psychological assessment” ) AND (judges OR legislation OR judiciary OR “Judicial System” OR court* OR magistrate* OR “legal system”) AND PUBYEAR > 2000 AND (LIMIT-TO (LANGUAGE, “English”) OR LIMIT-TO (LANGUAGE, “Spanish”) (“domestic abuse” OR “spouse abuse” OR “intimate partner violence”) AND (“mental health” OR “mental disorders”) AND (“criminal law” OR legislation)[MeSH Major Topic]) OR ((“battered women” [Title/Abstract] OR “domestic violence” [Title/Abstract] OR “intimate partner violence” [Title/Abstract] OR “domestic abuse” [Title/Abstract] OR “family abuse” [Title/Abstract] OR “family violence” [Title/Abstract] OR “spousal assault” [Title/Abstract] OR “spousal abuse” [Title/Abstract] OR “spouse abuse”)[Title/Abstract] AND (“mental health” [Title/Abstract] OR “mental illness” [Title/Abstract] OR “mental disorders” [Title/Abstract] OR psychiatr*)[Title/Abstract] AND (judges[Title/Abstract] OR legislation[Title/Abstract] OR judiciary[Title/Abstract] OR “Judicial System” [Title/Abstract] OR “family court*” [Title/Abstract] OR magistrate*[Title/Abstract] OR “legal system” [Title/Abstract] OR “legal aspect” [Title/Abstract] OR “criminal law”)[Title/Abstract]) AND ((english[Filter] OR spanish[Filter]) AND (2000:2020[pdat])) Filters: English, Spanish (“domestic violence” OR “intimate partner violence” OR “domestic abuse” OR “family violence” OR “family abuse” OR “spouse abuse” OR “spousal abuse”)/p (“mental health” OR “mental disorders” OR “mental illness” OR psychiat!) (“family violence” OR “family abuse” OR “domestic violence” OR “domestic abuse” OR battered OR “intimate partner violence” OR “spouse abuse”) AND (mental OR psychiatr* OR trauma OR psycholog* OR victimization)

### Study Selection

**Figure 1.**
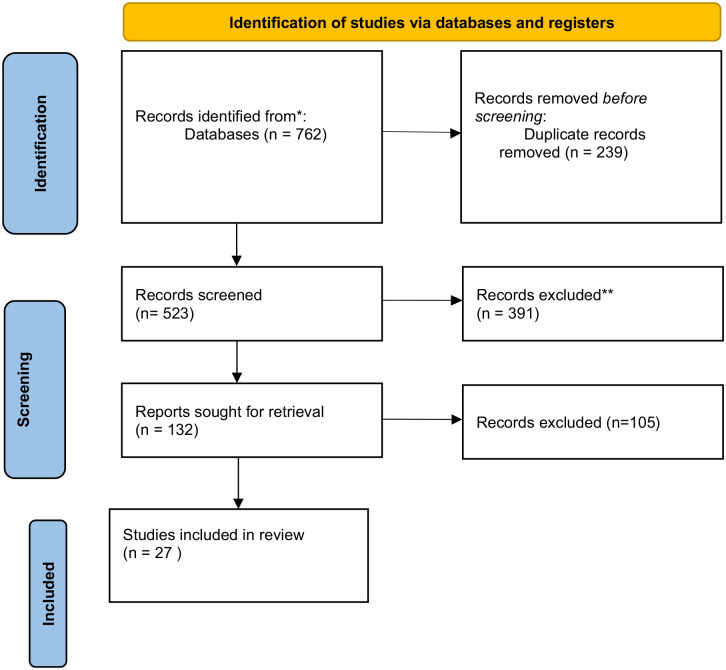
PRISMA flowchart illustrating inclusion of studies on scoping review of judicial understanding of the mental health impact of IPV on victims/survivors. *From*: Page et al. 2021.

These searches returned 762 potentially relevant studies, which were screened by title and abstract to ascertain whether they complied with the inclusion criteria. Initial screening of the articles resulted in the removal of 630 studies that did not meet the eligibility criteria. Two authors (JM, DH) independently reviewed the title and abstracts of the 132 remaining studies, resulting in removal of further 104 studies that, upon further examination, did not meet the inclusion criteria and were therefore excluded. The remaining 27 eligible studies were then retrieved and subjected to full-text review by two independent reviewers (PA, SHB) to determine the relevance of the research to the aims of this review. After the full-text review, including an interrogation of the reference list of each article and a further process of hand searching using Google and Google Scholar, all 27 studies remained eligible for inclusion (Figure 1).

### Data Extraction and Analysis

A data extraction template, constructed using a Microsoft Excel spreadsheet, (Microsoft, Redmond, Washington) was used to record relevant information such as purpose, research design, sampling method, sample characteristics, data collection method, method of data analysis, study results, limitations, and comments. Heterogeneity and the limited number of selected studies meant that statistical pooling of review results was not possible. Therefore, appropriate tables, figures, and narrative themes were developed to summarize the findings.

Thematic analysis techniques described by Braun and Clarke (2006) were used to code the publications. Coding involved reading and re-reading each publication and categorizing the text into emergent categories. Initially, each member individually read the publication and made notes about their initial impressions of the themes identified in the publication. After this first round of analysis, the team met together to collectively discuss and code themes as nodes within NVivo12 (Melbourne, Victoria, AUSTRALIA), which was used as a data management tool. Each researcher re-read each publication and coded them according to the themes that had been collectively established to guide the selection process.

## Findings

A total of 27 studies were included in the review (summarized in Table 1). The included studies were published between 2005 and 2023 in the United States (Bellew, 2005; Calton & Cattaneo, 2014; Cerulli et al., 2011; Crowe & Murray, 2015; Gilroy et al., 2015; Grossman, 2018; Logan & Cole, 2007; Logan et al., 2006; Nichols-Hadeed et al., 2012; Rizo et al., 2018), Australia (De Simone & Heward-Belle, 2020; Death et al., 2019; Douglas, 2018; Fitz-Gibbon et al., 2019; Jeffries et al., 2016; Roberts et al., 2015), Spain (Fariña et al., 2014; Regueira-Diéguez et al., 2015), United Kingdom (Hean et al., 2010), Iran (Rahnavardi et al., 2017) and Italy (Feresin, 2020). Most of the studies came from United States and Australia.

**Table 1.** Summary of Included Studies.

Author/s	Year	Country	Methodology	Purpose	Relevant Research Findings
Bellew	2005	USA	Qualitative study using in-depth interviews with ten professionals (including social workers and judicial actors/lawyers) from help-related Domestic Violence services and two survivors of Domestic Violence.	Explored the nature of abuse experienced by affluent survivors of Intimate partner violence (IPV) and the unique barriers that their economic privilege creates in help-seeking.	Economically privileged women experience unique barriers to accessing support services, such as severe psychological abuse, and lack of recognition of IPV due to their social & financial status which makes them less likely to access support.
Calton and Cattaneo	2014	USA	A longitudinal mixed-methods study using quantitative surveys and in-depth interviews with a sample of 142 survivors of IPV seeking help from the criminal justice system.	Explored the link between survivors’ perceptions of procedural/distributive justice and how it affected their mental health and willingness to use the system again in the future.	Survivors who perceived a fair/just court process experienced improved quality of life & mental wellbeing and an increased likelihood of seeking help in the future.
Cerulli et al.	2011	USA	A quantitative study using surveys on physical health, mental health, and social functioning of 95 female petitioners at an upstate New York Domestic Violence Intensive Intervention Court.	Explored collaborative work between legal and mental health services to assist IPV survivors with mental & physical health.	There are significantly high rates of mental health problems for IPV survivors which highlights as need for collaboration between legal and mental health services to support survivors.
Crowe and Murray	2015	USA	A mixed-method study using surveys and interviews with 231 survivors of abusive relationships.	Examined the stigma survivors of IPV experience from professional helpers.	Survivors felt significantly stigmatized by professional helpers, including judges (most commonly occurring in the court setting) which negatively impacted their mental wellbeing. It included feelings of being blamed, dismissed, or denied.
Death et al.	2019	Australia	A retrospective qualitative analysis of 357 publicly available judgments of the Family Court of Australia between 2010 and 2015.	Explored how parental alienation is used in the court setting in child sexual abuse cases.	One of five main themes “mothers as mentally ill” found that mothers’ mental health often impacts custody for mothers and the best interest of the child—highlighting the complex nature of dealing with child sexual abuse cases in a court setting.
De Simone and Heward-Belle	2020	Australia	A mixed-methods study that reports on data and observations from the authors’ professional experience of families experiencing domestic and family violence who have been reported to the Department of Child Safety, Youth and Women in Queensland.	Examined the institutional response and common representations of women who are mothering in the context of domestic violence which is prevalent within child-protection services.	This study found that the presentation of survivors impacts judicial response and that through engaging in domestic violence-informed capacity-building activities, child-protection workers and legal actors can redress gender-blind hegemonic practices in the child-protection space.
Douglas	2018	Australia	A qualitative study using interviews with 65 women who had experienced DFV and engaged with the legal system.	Examined a women’s long-term experiences in engaging with multiple legal processes (associated with domestic and family violence).	Engaging with multiple legal processes can negatively impact women’s health, including increased stress and trauma. Also highlights that some women hide mental health concerns over a fear of negative court outcomes.
Fariña et al.	2014	Spain	A quantitative study using a psychometric instrument with 105 Caucasian Spanish women.	Examined the Spanish standard of proof for forensic examination of psychological injury in judicial cases relating to IPV.	Standard forensic procedures for examining psychological injury because of IPV were not found to constitute valid proof for judges who acquitted defendants on the grounds of not guilty due to lack of evidence of psychological injury.
Feresin	2020	Italy	A qualitative study using semi-structured interviews with separated mothers who were IPV victims, and with social workers and psychologists/psychiatrists designated by courts to evaluate parenting skills. Expert reports, psychological assessments and legal documents were also analyzed.	Focused on parental alienation and explores women’s experiences as well as legal and social services’ practices in child custody cases	Professionals endorsed parental alienation and considered it a “feminine problem.” Women were often blamed and labeled as “engaging in parental alienation” when they were trying to ensure their children’s safety. Children’s accounts were interpreted as being a result of their mothers’ manipulation. Fathers were treated as victims of vindictive women who want to keep children to themselves. Men’s violent behaviors were not considered, and their role as fathers was seen as “inviolable.”
Fitz-Gibbon et al.	2019	Australia	Mixed-methods study drawing from a wider research project on family violence risk, which engaged over 1000 members of Victoria’s family violence system.	Examined the perceptions of Victorian family-violence practitioners on current practices and future needs for reform to improve family violence risk assessment for children.	Reinforces the need for specialist training and collaboration across services in supporting children impacted by family violence for example, emotional impact. Highlights the need for caution among practitioners, to avoid blame for mothers who are impacted by family violence.
Gilroy et al.	2015	USA	A qualitative study using interviews with 300 mothers who had experienced physical or sexual IPV and were seeking services for the first time at either a safe shelter for abused women or applying for a protection order.	Identified characteristics of women that affect access and use of community agencies.	Individualized attention was beneficial to help support women who have experienced IPV access and used community agencies, including specialized counseling services, emergency housing services, police, law enforcement, and legal services including applying to court for protection orders, faith community services, social services, crisis telephone hotline services and health care.
Grossman	2018	USA	This article reviews the approaches taken by various USA states on Domestic violence and Sexual Assault Cases.	Focused on the concept of unavailability in the court setting, why it occurs, its impact and how to combat its occurrence.	Mental illness and its influence on unavailability need to be recognized. This has an impact on both unavailability and the justice system.
Hean et al.	2010	UK	A quantitative study using proformas completed by a women’s specialist worker for 86 women defendants.	Identified women’s needs with the court system before entering prison (specifically through the Criminal Justice System).	Women attending court often have mental health issues that have not been addressed prior due to the stigma around being labeled mentally ill and its impact on the survivor.
Jeffries et al.	2016	Australia	Qualitative study using focus groups of 56	This qualitative study reporting on findings from a focus group aimed to gain perspective from professionals working in the court setting (family law) and how the health of the survivor is incorporated in the family reports (documents made by social workers and psychologists to provide evidence to the judge).	Family report writers often invalidated coercive control and ignored domestic violence, with a focus on trying to maintain the perpetrator’s relationship with their child.
Logan and Cole	2007	USA	Qualitative study using interviews with 662 women who received a protective order against a violent partner.	Examined the impact of stalking victimization on mental health and protective order outcomes (over the long-term).	Stalking is a significant risk factor for different forms of IPV and negative mental health outcomes, and court services often do not consider this when dealing with survivors.
Logan et al.	2006	USA	Qualitative study using interviews with 152 key informants.	Looked at the impact IPV had on women’s mental health outcomes and help-seeking.	Protections offered by protective orders are not often adequate for women who are experiencing stalking, with stalking negatively affecting women’s mental health.
Logan et al.	2006	USA	A mixed-methods study using interviews and surveys with 757 women who were enrolled in a study of women with protective orders against a male intimate partner.	Examined effective approaches to dealing with stalking survivors (in the justice and services settings).	Found that services do not recognize stalking as different from partner violence or the impact that stalking has on the mental health of survivors. Training is needed to improve approaches to help women who experience it.
Nichols-Haddeed et al.	2012	USA	A mixed-methods study using interviews with 190 women filing for a Protection Order against an intimate partner and archival review of court petitions.	Investigated how effectively court-ordered protection orders communicate survivors’ current danger and future risk of violence.	Judges may be inadequately prepared to make decisions that effectively improve survivor safety due to the absence of standardized risk assessments and an understanding of IPV dynamics. Evidence-based tools such as the Danger Assessment could be employed by judges to inform protection orders and improve survivor safety.
Rahnavard et al.	2017	Iran	A quantitative study using questionnaires with 110 women referring to family courts in Rasht during the year 2014.	Investigated types and causes of IPV among Iranian women and examines coping strategies to deal with it as identified by survivors.	Psychological abuse was identified as the most common form of violence and survivors identified “logical talk” as a good strategy for reducing IPV.
Regueira-Diéguez et al.	2015	Spain	A quantitative study that retrospectively analyses 582 case files classified as gender violence from the prosecutor’s office of Santiago de Compostela.	A retrospective analysis of cases of non-fatal IPV, aimed at improving understanding of the experiences of IPV of women in Spain.	Highlights the variability of the nature of IPV and its impact on mental wellbeing. There is a need for standardized concepts of IPV as well as for promoting greater understanding and assessment of IPV among health professionals and legal practitioners.
Rizo et al.	2018	USA	A quantitative study using questionnaires of 70 participants who completed the Mothers Overcoming Violence Through Education and Empowerment program.	An assessment of the effectiveness of a novel 13-week intervention aimed at addressing safety, parenting and mental health for mothers who were also survivors of IPV.	Female IPV survivors who are the primary caregivers of children are often mandated to engage with services. Through engagement with specialist group-based intervention aimed at addressing their mental health needs, IPV survivors saw significant improvement in symptoms of depression and post-traumatic stress.
Roberts et al.	2015	Australia	Qualitative study using interviews with 15 women who had left abusive relationships and who had, or were currently, engaged with the Federal Family Court process.	Identified the psychological impact engaging with the court system has on survivors and its influence on the family court process.	Engaging with the court can cause significant distress for survivors (including confronting their abusive partner in court) and experience difficulties engaging with the court or legal professionals, including experiencing feelings of a lack of empathy, invalidation and re-traumatized.
Romain Dagenhard	2020	USA	A critical discourse analysis approach was used to analyze 100 observations of probation review hearings in three domestic violence courts	Examined how judges, probation agents, attorneys, and probationers construct a probationer’s non-compliance based upon gender and racial discourses.	Gender differences in parenting responsibilities, mental health, and domestic violence discourses emerged, with racial differences in responsibility and mental health discourses.
Wallin and Durfee	2020	USA	A quantitative study that analyzed a sample of 580 PO filings from the DV protection order database.	Analyzed judicial and petitioner decision-making relating to firearm removal in cases of civil DV Protection Orders in Arizona.	Of 580 petitioners who were granted a protection order and requested firearm removal, only 50.1% were granted. Firearm removal was more likely to be granted in cases involving physical violence and threats to kill, however, was less likely to be granted in cases whereby the respondent is alleged to have mental health issues.
Woodhead et al.	2015	New Zealand	A qualitative study that analyzed 110 Family Court decisions by judges that involved applications for care or contact by parents of children 4 years or younger.	Examined judicial decision-making relating to post-parental separation care arrangements for children under 4 years old.	Overnight contact with the non-resident parent was the most likely care arrangement to be ordered, particularly among children who had an older sibling. Factors such as parental mental health, substance use, and domestic violence were not influential in care arrangements.
Woerner et al.	2023	USA	A quantitative study involving 298 women and data was collected by trained interviewers on questionnaire.	Investigated women’s court experiences, mental health, and willingness to engage the system in the future for IPV with a primary focus on the role of prior abusive relationships.	Women who had a prior abusive relationship were less likely to have contact with a court-based victim advocate, but there were no differences in the criminal order of protection level of restriction they requested. Women with prior abusive relationships reported greater depression symptoms and perceived stress, and less willingness to engage the system in the future. No differences emerged in post-traumatic stress disorder (PTSD) symptoms.
Wright and Johnson	2012	USA	Qualitative study using interviews with 111 women from shelter residents who reported experiencing IPV in their most recent relationship.	Examined the therapeutic effects for survivors after having a Protection Order issued on the abuser.	Although research is still lacking, it was found after CPO was issued survivors showed improved mental outcomes, including lower PTSD symptoms.

Studies used qualitative (Bellew, 2005; Death et al., 2019; Douglas, 2018; Gilroy et al., 2015; Jeffries et al., 2016; Logan & Cole, 2007; Logan et al., 2006; Roberts et al., 2015; Romain Dagenhardt, 2020; Woodhead et al., 2015; Wright & Johnson, 2012), quantitative (Cerulli et al., 2011; Fariña et al., 2014; Feresin, 2020; Hean et al., 2010; Rahnavardi et al., 2017; Regueira-Diéguez et al., 2015; Rizo et al., 2018; Wallin & Durfee, 2020; Woerner et al., 2023) and mixed methods approach (Calton & Cattaneo, 2014; Crowe & Murray, 2015; De Simone & Heward-Belle, 2020; Fitz-Gibbon et al., 2019; Nichols-Hadeed et al., 2012). Data was collected through individual interviews (Bellew, 2005; Calton & Cattaneo, 2014; Crowe & Murray, 2015; Douglas, 2018; Feresin, 2020; Gilroy et al., 2015; Logan & Cole, 2007; Logan et al., 2006; Nichols-Hadeed et al., 2012; Roberts et al., 2015), focus group discussion (Jeffries et al., 2016), surveys (Calton & Cattaneo, 2014; Cerulli et al., 2011; Crowe & Murray, 2015; Fariña et al., 2014; Rahnavardi et al., 2017), and document review (Death et al., 2019; Hean et al., 2010; Regueira-Diéguez et al., 2015; Romain Dagenhardt, 2020).

Thematic analysis uncovered five main themes that illuminated key areas, including: awareness of survivors’ experiences, gap in judicial actors’ knowledge, understanding of perpetrator tactics and risk factors, risks associated with disclosing mental health problems, training, and guidance (see Table 2). The studies demonstrate that there is a significant limitation or a gap in the literature exploring judicial actors’ awareness of IPV. The limited research available highlights significant gaps in judicial actors’ understanding of this issue and recommends strategies to increase their awareness and understanding.

**Table 2.** Themes Identified in the Studies.

Awareness of survivors’ experiences	Cerulli et al. (2011), Calton and Cattaneo (2014), Crowe and Murray, (2015), Death et al. (2019), De Simone and Heward-Belle (2020), Douglas (2018), Fariña et al. (2014), Feresin (2020), Gilroy et al. (2015), Grossman (2018), Hean et al. (2010), Rahnavard et al. (2017), Regueira-Diéguez et al. (2015), Rizo et al. (2018), Roberts et al. (2015), Romain Dagenhard (2020), Wallin and Durfee (2020), Wright and Johnson (2012)
Gap in judicial actors’ knowledge	Bellew (2005), Cerulli et al. (2011), Death et al. (2019), De Simone and Heward-Belle (2020), Douglas (2018), Feresin (2020), Jeffries et al. (2016), Logan and Cole (2007), Logan et al. (2006), Nichols-Haddeed et al. (2012), Regueira-Diéguez et al. (2015), Roberts et al. (2015), Romain Dagenhard (2020), Wallin and Durfee (2020), Woodhead et al. (2015), Woerner et al. (2023)
Understanding of perpetrator tactics and risk factors	Cerulli et al. (2011), Crowe and Murray (2015), Death et al. (2019), De Simone and Heward-Belle (2020), Douglas (2018), Feresin (2020), Gilroy et al. (2015), Hean et al. (2010), Jeffries et al. (2016), Logan and Cole (2007), Logan et al. (2006), Regueira-Diéguez et al. (2015), Romain Dagenhard (2020), Woerner et al. (2023)
Disclosing mental health problems	Bellew (2005), Cerulli et al. (2011), Crowe and Murray (2015), Douglas (2018), Fariña et al. (2014), Feresin (2020), Fitz-Gibbon et al. (2019), Gilroy et al. (2015), Grossman (2018), Hean et al. (2010), Nichols-Haddeed et al. (2012), Rahnavard et al. (2017), Regueira-Diéguez et al. (2015), Rizo et al. (2018), Woerner et al. (2023)
Training, and guidance	Bellew (2005), Calton and Cattaneo (2014), Crowe and Murray (2015), De Simone and Heward-Belle (2020), Fariña et al. (2014), Fitz-Gibbon et al. (2019), Jeffries et al. (2016), Logan and Cole (2007), Logan et al. (2006), Nichols-Haddeed et al. (2012), Regueira-Diéguez et al. (2015); Roberts et al. (2015)

### Awareness of Survivors’ Experiences

Twelve studies illuminated judicial actors’ understanding of the mental health impacts of IPV from the perspective of survivors who had been involved in the justice system (Calton & Cattaneo, 2014; Cerulli et al., 2011; Crowe & Murray, 2015; Death et al., 2019; Douglas, 2018; Feresin, 2020; Gilroy et al., 2015; Rahnavardi et al., 2017; Rizo et al., 2018; Roberts et al., 2015; Romain Dagenhardt, 2020; Wright & Johnson, 2012). The findings suggest that IPV survivors seek legal help for numerous issues, including seeking safety and protection, family law matters, child welfare, and criminal justice. Women in these studies felt that the impact of IPV to their mental health was poorly understood, misrepresented, and used to undermine their experiences, leading to not only failures to protect them, but resulted in actions that compounded trauma for them (Feresin, 2020; Roberts et al., 2015). They frequently reported experiencing invalidation and re-traumatization when navigating the family court system due to a lack of empathy and understanding from judicial actors (Bellew, 2005; Roberts et al., 2015). Women who sought help from legal services also reported further mental health impacts and re-traumatization (Feresin, 2020; Gilroy et al., 2015). Race, gender, socioeconomic situations, and mental health conditions also influenced how women were treated, with those affected by these issues facing stereotypes, judgmental attitude, and unfair treatment (Bellew, 2005; Feresin, 2020; Grossman, 2018; Hean et al., 2010). However, there were some positive examples and judicial actors that were IPV-informed frequently mandated interventions that focused on survivors’ mental health challenges and resulted in significant improvements to their mental health (Rizo et al., 2018). In situations where judges listened to survivors’ accounts and treated their evidence as equally important to the perpetrators, IPV survivors felt validated, respected, and listened to, even if the outcome was not in their favor (Calton & Cattaneo, 2014; Wright & Johnson, 2012). However, there were not many examples of this, and most studies argued that the legal system must work toward acknowledging mental distress as a significant factor that can interfere with a survivor’s ability to testify, arguing that appropriate steps should be taken to ensure that victims/survivors can testify (Grossman, 2018).

### The Gap in Judicial Actors’ Knowledge

Several studies demonstrated the need for judicial actors to have a greater awareness and understanding of the gendered nature of IPV (Cerulli et al., 2011; De Simone & Heward-Belle, 2020; Douglas, 2018; Hean et al., 2010; Jeffries et al., 2016; Logan & Cole, 2007; Logan et al., 2006; Nichols-Hadeed et al., 2012; Wallin & Durfee, 2020). There are often misconceptions related to the role of gender in IPV. Sexist perceptions of women as histrionic or provocative can decontextualize their responses to IPV, and result in judicial decisions that fail to hold perpetrators of IPV accountable for their crimes (Woodhead et al., 2015). Included studies underscored how judicial actors can better meet the needs of survivors of IPV and hold perpetrators accountable by recognizing and addressing gender-based assumptions, misconceptions, and misunderstandings (Bellew, 2005; Woodhead et al., 2015). Other studies identified a striking gender bias in family court proceedings (Death et al., 2019; Feresin, 2020; Romain Dagenhardt, 2020). For example, in Death et al.’s (2019) study, maternal mental illness was more frequently used (in one-third of cases) to invalidate mothers’ allegations of child abuse and to remove contact and/or custody, in comparison to paternal mental illness (used in just 2% of cases). Women with better socioeconomic conditions were treated unfairly and not seen as victims (Bellew, 2005). In addition, stereotypical, judgmental, and biased attitude was experienced by victims/survivors (Douglas, 2018; Romain Dagenhardt, 2020).

### Understanding of Perpetrator Tactics and Risk Factors

Although some studies found that some judges possess an adequate level of understanding of IPV risk factors, there is a significant lack of understanding in others. For example, American judges were more likely to grant the removal of firearms when Protection Order petitions contained elements of violence, death threats, and claims that the respondent owned a gun (Wallin & Durfee, 2020). This suggests that some judges recognize lethality risk factors. However, other researchers found that when making decisions about protection order and custody issue, vital information such as perpetrators’ substance misuse, suicidal threats, and use of sexualized violence was not considered (Logan & Cole, 2007; Nichols-Hadeed et al., 2012; Woerner et al., 2023) and litigation coercion was not well understood by judicial actors (Bellew, 2005). Perpetrators with access to financial resources frequently used the courts and legal processes to maintain power, control and to harass partners, and ex-partners. Coercion through litigation deleteriously impacted survivors’ finances and mental health and judicial actors did not understand it as an instrumental tactic of coercive control. This lack of understanding can compound women’s and children’s trauma, decrease their safety, and deter them from seeking future help. Additional concerns raised include the presence of stereotypical and biased attitudes toward survivors of diverse backgrounds, particularly women.

### Risks Associated with Disclosing Mental Health Problems

Several studies demonstrated a concerning phenomenon whereby victims/survivors are reluctant or are advised by legal representatives not to disclose IPV and its impact to their mental health. This occurrence has been associated with multiple factors, including fears that they will be blamed or stigmatized within the legal system and experience adverse legal outcomes. Cerulli et al. (2011) reported high rates of mental health problems among a cohort of victims/survivors seeking protection orders in the United States. They found that advocates frequently discouraged survivors from seeking mental health services during the legal process due to fears that stigma and misunderstandings would lead to victim-blaming and adverse outcomes. Similarly, another study reported that IPV survivors who experienced mental health issues experienced stigma, blame, and minimization of their experiences from professionals, family, and friends, which was exacerbated when people did not understand the impact of IPV (Crowe & Murray, 2015).

The idea that women survivors think and act strategically in their legal cases and may be reluctant to share mental health problems and treatments with their legal team is supported by Douglas’ (2018) research. Several studies demonstrated a sexist double standard in the legal system whereby violent fathers with mental health issues are frequently granted contact with their children, whereas mothers with mental health issues are perceived to be delusional or overprotective and often denied contact (Douglas, 2018; Feresin, 2020; Romain Dagenhardt, 2020; Woerner et al., 2023). Women’s experiences of IPV are often invalidated, and the focus is on trying to maintain the perpetrator’s relationship with his children (Feresin, 2020; Hean et al., 2010; Jeffries et al., 2016).

De Simone and Heward-Belle (2020) found that the representation of IPV survivors who experienced mental health problems had a considerable influence on judicial care and protection decisions. The authors argued that judicial actors frequently failed to understand the relationship between mental health, IPV, and access to justice. Judicial actors frequently represented mothers and fathers differently, perpetuating gendered social norms that inequitably hold mothers solely responsible for the care and protection of children (Death et al., 2019). Therefore, judicial actors frequently portrayed women as “failing to protect” children from men’s violence, while simultaneously absolving violent fathers from the responsibility of their actions and its impact on their children.

### Training and Guidance

Several studies identified that the need for increased training and guidance for judicial actors and other professionals, particularly around the mental health impacts of IPV across multiple jurisdictions, is an important step in improving judicial responses to survivors of IPV (De Simone & Heward-Belle, 2020; Fariña et al., 2014; Fitz-Gibbon et al., 2019; Logan et al., 2006; Nichols-Hadeed et al., 2012; Regueira-Diéguez et al., 2015; Roberts et al., 2015). Several studies suggest that judicial training and capacity building activities could radically improve responses to victim/survivors and their children (Bellew, 2005). Topics to be covered include general information on the dynamics and impact of IPV on adult and child survivors (Feresin, 2020), the effectiveness of risk assessment procedures and tools (Fariña et al., 2014; Fitz-Gibbon, 2019; Regueira-Diéguez et al., 2015; Nichols-Haddeed et al., 2012), perpetrator tactics and their impact (Bellew, 2005; Nichols-Haddeed et al., 2012; Regueira-Diéguez et al., 2015), the intersections between IPV, substance misuse and mental health and trauma-informed judicial responses (Bellew,2005; Regueira-Diéguez et al. 2015; Romain Dagenhardt, 2020), and importance of understanding bias (Romain Dagenhardt, 2020).

## Discussion

The purpose of this scoping review was to consider what is known from contemporary scientific research about judicial actors’ understanding of the mental health impacts of IPV on survivors (from the perspective of survivors or judicial actors) and what strategies and recommendations have been made to increase judicial actors’ awareness of these impacts. This review highlighted that survivors experience numerous challenges within the legal system, particularly when they experience mental health issues arising from IPV. The intersectionality of IPV survivors’ experiences—encompassing gender, race, socioeconomic status, and more—further complicates their journey through the legal system. In many jurisdictions, the legal system acts as the most powerful institution that survivors encounter and the ramifications of this can have long-lasting impacts on multiple domains of their lives, including safety and protection from future violence, family law issues, child welfare, and mental health. Considering the strong correlation between IPV and experiences of mental distress, anxiety, and symptoms of PTSD (Fanslow & Robinson, 2004; Gulliver & Fanslow, 2013), it is essential that judicial actors, particularly judges, can recognize and respond appropriately to survivors who are encountering the judicial system. Their comprehension of the mental health repercussions of IPV must transcend mere recognition, evolving into empathetic engagement that informs both legal reasoning and the provision of support services, thereby mitigating the risk of secondary victimization.

The findings of this review point toward an overall limited understanding among judicial officers and judges about IPV. Traditional gender-based assumptions continue to infiltrate judicial responses to women experiencing IPV, including around their supposed emotional “volatility” and responsibilities as mothers in caring for and “protecting” their children from violence (Hamel, 2018; Roberts et al., 2015). Factors including alcohol abuse and mental health problems are used by judges to excuse, minimize and justify violent male behavior, particularly sexual offending, and simultaneously to cast doubt on women’s testimonies of abuse (Coates & Wade, 2004, 2007).

Negative experiences within the justice system are likely to influence survivors’ future help-seeking behaviors and their likelihood to access support. Further to this, the findings suggest that the complexity of the judicial system can exacerbate underlying mental health issues for victims/survivors, who may then be fearful about seeking help from the justice sector due to stigmatization relating to mental distress (Douglas, 2018). This review has demonstrated that survivors can be retraumatized throughout the judicial process, which can replicate the power imbalance and coercive control experienced by the victim/survivor within an abusive relationship (Douglas, 2018; Grossman, 2018). Survivors from marginalized communities may face additional barriers in the judicial process, including discrimination and biases that can affect the understanding and treatment of their cases. This can lead to disparities in outcomes, where some survivors’ experiences and mental health impacts are not adequately acknowledged or addressed. This also makes survivors reluctant from seeking any help from professionals in health and social care and judicial system.

The literature suggests that misconceptions and limited understandings of IPV within the legal system is a widespread problem occurring across multiple jurisdictions with studies from Australia, United States, New Zealand, Iran, Canada, Spain, and the United Kingdom. These studies drew attention to the array of complex issues and misconceptions within legal systems, which compound survivors’ ability to feel safe and which have a lasting impact on their mental health. A large majority of the studies focused on the experiences and perspective of survivors and not necessarily of judicial actors. Collectively, these studies provide evidence for the need to increase judicial actors’ awareness of IPV risk factors. By increasing judicial actors’ understanding of all risk factors associated with IPV and domestic homicide, judicial actors can better meet the safety needs of victim/survivors and their children by creating effective orders and holding perpetrators to account. These studies also highlight the need for research to understand perspective of judicial actors so appropriate strategies can be developed to enhance their understanding of the issue to help them make informed and better decisions to support survivors of abuse.

The review demonstrates how gendered social norms can further perpetuate the marginalization and disempowerment of women involved in judicial processes. It highlights the need for an intersectional approach to understanding and responding well to the complex needs of victims/survivors that addresses the interplay between IPV, mental distress, gender, and power dynamics. This will help judicial actors recognize the unique challenges faced by survivors from marginalized communities and ensuring that judicial responses are sensitive to these complexities. Overall, evidence highlights a crucial need to increase judicial actors understanding of the risks associated with disclosing the mental health consequences of IPV for victim/survivors and emphasizes the need for a more nuanced and equitable response from the legal system (Cerulli et al., 2011; Douglas, 2018; Hean et al., 2010).

The findings of the review identified strategies for improving judicial understandings of the complexity of IPV, and the many ways in which tactics of coercive control were employed by perpetrators. Recommendations included professional training of judicial actors to increase awareness of IPV, understanding of the impact of trauma, and the knowledge of the intersection between IPV and mental health issues. The integration of trauma-informed training programs for judicial actors can bridge the knowledge gap, fostering an environment where survivors feel understood, respected, and more confident in the pursuit of justice and healing. Improved judicial responses may lead to increased safety for survivors and limit the potential for further traumatization. Further to this, improving knowledge about IPV across wider society may result in improved community and service responses to survivors of IPV across multiple domains. Perlin and Gallagher (2017) argued that judges play a significant role in educating the community and raising awareness of problems in society such as IPV, thus highlighting the importance of judicial training and research to knowledge translation. As mentioned earlier, most of the studies focused on the perspective of survivors and there is a serious dearth of literature about judges, lawyers, and other judicial actors understanding of the mental health impacts of IPV; therefore, there is an urgent need to conduct specific research on these groups.

### Strengths and Limitations

A scoping review enable**s** a preliminary assessment of the potential size and scope of available research literature. It aims to identify the nature and extent of research evidence available on a given topic (Mak & Thomas, 2022; Peters et al., 2020). Although scoping reviews are useful in bringing evidence together, the reader needs to be aware of methodological limitations that apply. For instance, scoping reviews may lead to broader and less specific searches; multiple searches may be required as was the case with this review. A further limitation relates to the researchers’ decision to employ broad Boolean operator terms in relation to mental health. Although this is a common approach, if specific mental health disorders listed in the Diagnostic and Statistical Manual, Version 5 were used as search terms, the search would have yielded different results. However, the scoping review methodology was appropriate to elicit a broad array of published articles on an under-researched topic. Further research specific to different stakeholders’ experiences (e.g., perpetrators, health and welfare practitioners, children, and young people) would be useful.

## Conclusion

Examining contemporary scientific evidence in relation to judicial actors’ understanding of the mental health impacts of IPV on women survivors is key to developing a fair and responsive system. Table 3 documents key practice, policy and research implications arising from this scoping review that could lead to improved access to justice for women survivors. The findings suggest that misconceptions about IPV, trauma, and gender-based assumptions have an adverse impact upon survivors’ ability to achieve a sense of safety and well-being through the legal system.

**Table 3.** Implications for Practice, Policy, and Research.

Practice	• Increase judicial actors’ understating of the mental health impacts of intimate partner violence (IPV) by providing appropriate training and education. • Improve judicial actors’ practices to ensure that they are responsive to the safety and wellbeing needs of survivors. • Ensure that survivors feel safe to disclose IPV and the mental health impacts without fear of adverse outcomes due to stigmatization and/or discrimination. • Aid in the recognition of litigation coercion as a tactic of power and control used by perpetrators of IPV.
Policy	• Provide high-quality, evidence-based training within judicial programs and legal training at tertiary institutions to increase knowledge and skills of judicial actors to provide IPV-informed and trauma-informed responses. • Build capacity of judicial actors to improve quality of documentation presented to judicial decision-makers to ensure pattern of IPV and associated risks are accurately conveyed. • Design legal settings that are physically and emotionally safe for survivors
Research	• Conduct more research that investigates judicial actors’ understanding of the mental health impacts of IPV on survivors and how they apply their knowledge in practice. • Explore survivors’ perceptions of the impact of judicial training and other capacity building initiatives on their experiences within legal systems. • Initiate more research that investigates how intersecting aspects of survivors’ identities influence judicial outcomes.

There is a need for much greater professional training among judicial and legal actors, not only to deepen their understandings of IPV on a broader scale but also to develop a greater understanding of the mental health impacts of IPV on survivors. Collectively, findings indicate that since judges are making decisions based on the evidence that is put before them, legal actors within the overall court system—including lawyers who prepare documents need an education that highlights the mental health impacts of IPV on survivors. This would ensure that there is accurate evidence put before courts of the dynamics, severity, and impact of IPV, including the full gamut of tactics used by perpetrators and the connection between experiencing these tactics and mental health impacts.

A more thoughtful representation of survivors including their resistance to oppression, based on a comprehensive analysis of the perpetrators’ pattern of violence and coercive control provides judges with accurate evidence to make considered judgments that can underpin social responses that support women and children survivors. Adopting policies that prioritize survivors’ mental and emotional well-being in legal proceedings can transform the judicial system into a conduit for healing, rather than an arena of additional trauma. The ultimate aim is to cultivate a judicial landscape where the nuanced realities of IPV survivors are not just acknowledged but are central to the formulation of responses that uphold justice and facilitate recovery.
